# A73 MICROBIAL CONTRIBUTION TO THE RISK OF FUTURE CROHN’S DISEASE: STRATIFICATION BY SUBCLINICAL INFLAMMATION LEVELS MEASURED BY FECAL CALPROTECTIN

**DOI:** 10.1093/jcag/gwae059.073

**Published:** 2025-02-10

**Authors:** Q LI, W Turpin, P Olivera Sendra, M Xue, A Griffiths, R Panaccione, L Dieleman, A Steinhart, K Jacobson, S Lee, K Croitoru

**Affiliations:** Sinai Health, Toronto, ON, Canada; Sinai Health, Toronto, ON, Canada; Sinai Health, Toronto, ON, Canada; Sinai Health, Toronto, ON, Canada; The Hospital for Sick Children, Toronto, ON, Canada; University of Calgary, Calgary, AB, Canada; University of Alberta, Edmonton, AB, Canada; Sinai Health, Toronto, ON, Canada; BC Children’s Hospital, Vancouver, BC, Canada; Sinai Health, Toronto, ON, Canada; Sinai Health, Toronto, ON, Canada

## Abstract

**Background:**

Crohn’s disease (CD) prevalence is rising globally, but its pathogenesis remains unclear. Our group found specific microbes predict future CD risk in healthy first-degree relatives (FDR), whereas high fecal calprotectin (FCP) level (>100ug/g) was also significantly associated with a 6-fold higher risk of CD onset. However, the influence of FCP on how microbial communities contribute to CD risk remains unknown. Understanding microbial interactions with gut inflammation may offer insights into CD onset.

**Aims:**

This study investigates how microbial composition and function contribute to CD risk in healthy in association with their baseline FCP levels.

**Methods:**

Healthy FDRs from the CCC-GEM Project were followed for CD onset. Using baseline stool samples, microbial compositions were analyzed with 16S rRNA sequencing and functional predictions with PICRUSt2. Cox PH models assessed relationships between microbiome composition, functional pathways, and CD risk stratified by high or low levels of subclinical inflammation, defined by FCP with a cut-off of 100 ug/g (FCP_>100_ and FCP_<100_, respectively). q<0.1 was considered statistically significant.

**Results:**

We followed 3430 FDRs for a median of 7.7 years, among which 76 developed CD. In the subgroup with FCP_>100_ a higher abundance of *Ruminococcus torques group*, an undefined *Ruminococcus* genus, and *Romboutsia* were significantly associated with an increased risk of CD. In contrast, *Oscillospiraceae* family was consistently associated with a lower CD risk in both FCP_>100_ and FCP_<100_ subgroups.

Microbial functional pathways analysis revealed that peptidoglycan-N-acetylmuramic acid deacetylase, 4’-phosphopantetheinyl transferase, and iron(III) transport system ATP-binding protein were consistently associated with a decreased risk of CD in both FCP_>100_ and FCP_<100_ subgroups. However, pathways including Flagellar motor switch protein FliN and Carboxynorspermidine decarboxylase were associated with increased risk of CD, but only in the FCP_>100_ subgroup. In contrast, geranylgeranyl diphosphate synthase and heat shock protein HspR were associated with increased CD risk in the FCP_<100_ subgroup.

**Conclusions:**

The microbial contribution to CD risk in healthy FDRs may vary depending on baseline FCP levels. Specific taxa and functional pathways were identified as contributors to CD risk in FDRs in the presence or absence of intestinal inflammation. Notably, the *Oscillospiraceae* family was consistently associated with a decreased risk of CD, highlighting its potential as a therapeutic target in the pre-clinical phase of CD.

**Funding Agencies:**

**None**

